# Diphtheritic Paralysis in Children

**Published:** 1895-01-12

**Authors:** G. A. Sutherland

**Affiliations:** Physician to the North London Consumption Hospital, Registrar to the Paddington Green Children's Hospital


### Medical Progress and Hospital Clinics.

[*The Editor will be glad to receive offers of co-operation and contributions from members of the profession. All letters should be addressed to* The Editor, The Lodge, Porchester Square, London, W.]

### DIPHTHERITIC PARALYSIS IN CHILDREN.

By G. A. Sutherland, M.D., M.R.C.P., Physician to the North London Consumption Hospital, Registrar to the Paddington Green Children's Hospital.

(*Continued from page* 240.)

**Pathology.**—The exact pathology of this affection has not yet been determined, because no definite anatomical lesion is invariably present. The muscles have been found pale and atrophied, the nerves in a state of parenchymatous degeneration, and the central motor cells shrunken and presenting signs of granular and fatty degeneration. In many cases no change has been made out in nerves or cells. There appears to be some poison associated with the diphtheritic bacillus, possibly of the nature of a ptomaine, which is absorbed into the system and manifests itself some time after the acute disease has disappeared. This delayed action is difficult of explanation. It is held by some that this poison has a special affinity for the motor nerve cells, which are chemically or anatomically altered, and that the disturbance thus produced is shown in the parenchymatous degeneration of the peripheral nerves which ensues. The amount and virulence of the poison will determine the extent of the central lesion, and that again is revealed in the resulting paralysis. By others the primary change is held to be a peripheral neuritis, which commences in the nerves supplying the regions of membranous formation in diphtheria, and later on develops in other parts of the body.

**Treatment.**—The occurrence of diphtheritic paralysis is so frequent, and the consequences in some cases are so serious, that in every case of diphtheria precautionary measures ought to be taken. The most important precaution is the maintenance of the recumbent posture in bed for at least two weeks after all trace of membrane has disappeared, and the temperature has fallen to normal. Even then the patient is to be allowed to sit up only if there are no paralytic symptoms present and no evidence of cardiac weakness. He ought to be kept under observation for at least another month, and the parents warned as to the necessity for observing and reporting at once any difficulty in speaking or swallowing, or increased feebleness. Occasionally the development of the paralytic symptoms is delayed for one or two months, but in such cases convalescence does not progress satisfactorily, and an increasing amount of general feebleness is found if the patient is carefully examined. During the stage of convalescence from the primary attack it is advisable to continue the local disinfection of the throat, as cases have occurred in which Loeffler's bacilli were present in the crypts of the tonsils months after the subsidence of all symptoms of acute diphtheria.

On the occurrence of paralysis, however slight, absolute rest in bed must be insisted on. The patient is ordered to lie on his back on a mattress without any pillow, and if a young child his shoulders are to be fastened down so as to prevent the possibility of his sitting up in bed. Every effort, in short, must be made to avoid any voluntary muscular effort, which at all times reacts injuriously on the heart, and in advanced cases is a common cause of sudden cardiac failure. The bowels must be carefully regulated by laxatives or enemata, so as to prevent any straining at stool. Symptoms such as coughing, crying, or vomiting must be alleviated as speedily as possible. This absolute rest may appear at first sight a somewhat severe and difficult line of treatment in the case of children, but, as already pointed out, a condition of apathy is usually present, and the patient himself prefers to lie quite still, and is even disinclined to talk much.

The diet is to be as full and nourishing as is compatible with the patient's digestive powers. When there is a difficulty in swallowing, feeding by the stomach tube is beneficial, by means of a catheter introduced through the mouth or nose. In cases where sickness is present the food may be peptonised with advantage, and if toleration is not then established it is advisable to feed entirely by the rectum for a few days. This is usually well borne, provided that the amount administered each time is small (two or three ounces), and that the rectum is washed out once daily with warm water. Brandy is to be given when any symptoms of cardiac or respiratory weakness are present, and in advanced cases it must be employed in full doses. To a child of five years a drachm of brandy may be given every three hours, and it may be necessary to increase the dose to a drachm every hour. The drug which gives the most beneficial effect in diphtheritic paralysis is undoubtedly strychnine. It may be given by the mouth or preferably by hypodermic injection. To commence with, a dose of $\frac{1}{80}$ gr. may be ordered every six hours, or if the subcutaneous method is employed, $\frac{1}{10}$ gr. twice daily. The physiological effects of strychnine are not easily produced in this affection, and where the cardiac and respiratory centres are involved, the above doses may be considerably increased. The chief guide as to the amount is to be found in the condition of the heart and the respiration. Atropine in doses of $\frac{1}{200}$ gr. is recommended by some physicians in severe cases of paralysis, and by others belladonna is given all through the course of the disease. Galvanism has been employed, but although it may be of service in restoring the trunk and limb muscles in the convalescent stage, its value in the cardiac or respiratory crises is very questionable.

The duration of the treatment varies from one to three months from the onset of paralytic symptoms. The patient must not be allowed to sit up in bed until at least a week after the last trace of paralysis has gone. The return of the knee jerk may be delayed for some months after the disease has been cured, and so this symptom is not to be reckoned as one of the factors in determining the duration of treatment. The process of getting about again must be conducted very deliberately, as the effects of the illness on the heart are persistent. After the patient is able to walk about, a change to the seaside, moderate exercise, massage, and general tonics will speedily repair the wasted condition of the muscles, and restore the general health.

